# Application of Digital Informatics in Precision Prevention, Epidemiology, and Clinicogenomics Research to Advance Precision Healthcare

**DOI:** 10.1055/s-0044-1800753

**Published:** 2025-04-08

**Authors:** Qiang He, Patrick J. Silva, Marcia Ory, Ni Wang, Kenneth S. Ramos

**Affiliations:** 1 Texas A&M Health; 2 Department of Mechanical Engineering, Texas A&M University

**Keywords:** Clinicogenomics, Digital Informatics, Precision Prevention, Epidemiology, Public Health Informatics, Social Determinants of Health, Surveillance, Telehealth

## Abstract

**Objectives** : To summarize recent public health informatics and precision epidemiology developments impacting the healthcare ecosystem. The influence of new technologies and precision approaches in surveillance and management of chronic diseases is high-lighted as areas of clinical practice where digital informatics can markedly improve pop-ulation health.

**Methods** : In this narrative review, we summarized the main themes from research and practice to define disease prevention and public health trends. Publications on public health informatics and precision epidemiology were searched using Google Scholar us-ing the following keywords: “digital informatics”, “precision in prevention”, “precision epi-demiology”, “public health surveillance”, “clinicogenomics” and combinations thereof. In addition, we introduced the principles of a clinicogenomics registry as a case study to empower underrepresented communities and to reduce health disparities.

**Results** : Technology applications such as telehealth and digital information tools fre-quently intertwine with public health informatics and precision epidemiology in efforts to identify and target individuals and populations at risk of disease. There is an urgent need for more investigations and evaluation of the validity and utility of digital platforms, including artificial intelligence (AI) and predictive analytics to advance precision preven-tion and epidemiology. The major precision-based opportunities identified included: (1) the utilization of digital tools, (2) a public health strategic framework, (3) tele-health/telemonitoring tools, (4) digital twins to simulate and optimize care models, (5) clinicogenomics registries, (6) biomarker analyses and omics panels, and (7) mobile health.

**Conclusions** : Successful implementation of precision prevention and epidemiology ini-tiatives requires development of a researcher and practitioner workforce that is well-versed in informatics and public health. The positive impact of precision healthcare ap-proaches depends on solutions and technologies that connect digital patient information with wearable devices, mobile apps, telehealth, and digital analytics using AI. The vital components required to successfully integrate public health informatics, precision pre-vention and epidemiology are people, data, and tool systems, albeit within legal and ethical constraints. Together, these applications can significantly improve actionability of public health surveillance and societal trends in the preservation of health and disease prevention.

## 1. Introduction

**Figure 1.**
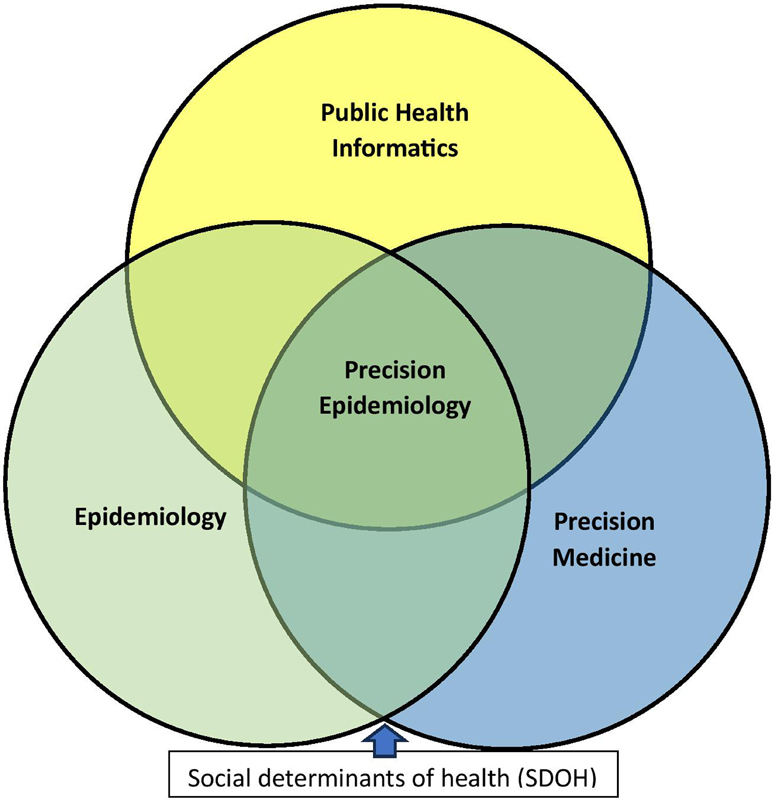
Disciplinary Interrelationships in the Precision Healthcare Ecosystem.

Informatics harnesses the power and possibility of digital technology and transforms data and information into knowledge and insights. Public health informatics and epidemiology are closely related, as both fields of study rely upon the use of data and technology to improve public health outcomes. Public health informatics is the application of information technology and computer science to public health practice, research, and learning [ 1 ]. Epidemiology studies the distribution and determinants of health and disease in populations and applies these findings to population health management [ 2 ]. Among new developments in epidemiology, precision prevention is a new concept and practice aiming to prevent chronic or infectious diseases by identifying and targeting individuals at high risk of developing disease. Precision epidemiology [ 3 ] is an emerging field that uses genomic technologies to provide information on molecular diagnosis and treatment regimens at both the individual and population levels. It is a multidisciplinary field ( Figure 1 ) that uses genomics, big data, and machine learning (ML)/AI to predict health risks and outcomes and to improve health at the population level [ 4 ]. Providing guidance for public health action, social determinants of health (SDOH) are recognized as a key underlying contextual framework for precision healthcare [ 5 ]. SDOH are non-medical factors that influence health outcomes and that, importantly, constitute the basis for health inequities in income, education, employment, and/or social support structures. These factors define the social environment of a specific individual and significantly contribute to population level changes [ 6 ].

The COVID-19 pandemic is emblematic of the huge impact of genomics technologies and public health informatics in medicine and public health [ 7 ]. These relationships were summarized by Yu et al. [ 8 ] in their study of the utility of COVID-19 databases and open access knowledge management systems for public health actions. The pandemic also brought to the forefront the importance of curated data resources in building public policy and practice. Public health informatics leveraged predictive analytics and big data to better understand the COVID-19 pandemic and generated deliverables used to inform healthcare professionals, policymakers, government agencies, and the public [ 9 ]. Researchers and healthcare professionals used informatics tools and principles to develop solutions for surveillance, outbreak or cluster recognition and response, acquisition of laboratory information, field investigation and mitigation strategies. Public health information systems optimally supported standardization of functions, though much work remains to be done to bring uniformity to these processes.

Data sharing tools and cloud computing have given medical researchers and healthcare providers tools, insights, and knowledge not previously available. For example, in the fight against COVID-19, the availability of large research communities with shared informatics tools proved to be pivotal to the rapid analysis of public health data and the creation of dashboards, maps, and interactive, audience-specific communications. These capabilities provided the public and other stakeholders with nearly real-time information on disease prevalence, morbidity and mortality statistics, and vaccination availability. The tools of precision epidemiology helped to explain individual variability in COVID-19 symptoms and enabled a more targeted approach toward infectious disease control.

While public health informatics helped to generate insights quickly and accurately based on actual healthcare data and real-world evidence (RWE), a complete picture of the patient journey and the waxing and waning of the pandemic at the population level remained beyond the reach of public health databases [ 10 ]. It is now more broadly recognized that efforts to integrate SDOH domains into electronic health records (EHRs) can help with tracking of specific outcomes to improve timely and actionable risk prediction [ 6 ].

Digital informatics technology has significantly changed many areas of modern life, including electronic health (e-health). In the late 1990s, e-health was introduced to convey the promise and potential of applying e-commerce to the health sector [ 11 ] and to document the patient journey beyond the walls of the clinic or the provider encounter. Digital health actively uses cloud-based data collection technologies (e.g., ambient listening, wearable sensors, mobile computing) to improve public health [ 12 ]. A rise in the availability and adoption of devices (e.g., smartphones, tablets, smartwatches) is driving a move towards mobile health (mHealth) [ 13 ]. Ambient data have promising potential to enhance public health surveillance and response, allowing for more refined stratification of population health-related data by time, place, and personal characteristics. It potentially allows greater “precision” in the public health response by enabling internet-based platforms, social media, ML, and predictive analytics [ 14 ].

In addition to illustrating the use cases for one or more of the emerging technologies in public health domains, this review addresses current deficiencies. It provides insights into critical advances in precision prevention and epidemiology and the overall context of open problems and opportunities in these fields. Our focus is on public health informatics and its impact on precision healthcare using COVID-19 and chronic diseases as case examples.

## 2. Methodology

The review was focused on published scientific papers and literature obtained from agency websites, such as the Centers for Disease Control and Prevention (CDC). The final product was not envisioned as a systematic review, but rather a landscape assessment of the field combined with perspectives and projections for the future. Most of the publications cited were published within the 4 -year range (January 2020 to December 2023). Papers were searched through Google Scholar, with keywords including “digital informatics”, “precision in prevention”, “precision epidemiology”, “public health surveillance”, “clinicogenomics”, and combinations thereof. Google Scholar was utilized because it provides more extensive coverage than PubMed. Manual curation was implemented in the final step by selecting the most appropriate studies and the references cited therein, followed by a final review of overlap with the MEDLINE database. The final listing included 58 papers and websites selected from more than 2,000 publications initially identified in the Google Scholar search. Approximately 100 review-type articles focused on precision public health, societal trends in health preservation, disease precision prevention, were also reviewed.

## 3. Results

### 3.1. Precision Public Health

As noted earlier, precision epidemiology is a field of study and practice that couples population-scale genomic data and computational approaches to elucidate individual and population level variations in health risks and outcomes. Public health surveillance is the ongoing, systematic collection, analysis, interpretation, and dissemination of health data to inform public health action across time and geography. The latter is a critical component of public health practice as it enables the detection of disease outbreaks, the identification of individual, environmental, and socio-economic risk factors for disease, and the evaluation of public health interventions. The Office of Genomics and Precision Public Health at the CDC integrates advances in genomics and precision health into public health research, policy, and programs. Advances in precision public health will fuel a shift away from disease-centered care, as described by Canfell et al. [ 15 ] in a study of anticipated shifts from disease-centered care to consumer-centered health showing that social, environmental, and behavioral health determinants explain 70% of health variance. Such shifts will continue as the global syndemic of preventable chronic diseases further strains healthcare systems.

Beyond genomics, granular data derived from population surveillance are being used to design targeted public health interventions. Big data (including traditional public health data, cell phone mobility data, wearable fitness trackers data, geographic information, sewage data indicating intensity and distribution of SARS-CoV-2 virus), digital technologies, and mobile health applications have been instrumental in defining the natural history of disease and identifying prognostic factors through ML and AI [ 16 ]. Twohig et al. [ 17 ] completed a cohort study comparing SARS-CoV-2 delta (B.1.617.2) hospital admissions and emergency care attendance risk to alpha (B.1.1.7) variants. This study generated one of the largest whole-genome-sequencing datasets for SARS-CoV-2 in England for 2021. The dataset included individual-level data of all laboratory-confirmed COVID-19 cases, up to 98% of hospital activity, and all vaccinated individuals registered with a general practitioner. Patients infected with the delta variant showed greater than a two-fold increased risk of hospital admission compared to those with the alpha variant. Thus, the use of precision epidemiology coupled with the tools of public health informatics and individual and population level omics' data can be used to improve public health outcomes and to formulate public health recommendations. This is evidenced by the identification of sex , age, sickle cell anemia, cancer, severe asthma, and pregnancy as risks factors for severe COVID-19 infection using these approaches [ 18 ].

The dawn of digital health entails cloud and mobile computing to enable the digital citizen to initiate a precision epidemiology study where the research subject can proactively contribute to documenting his/her own journey. A new care model can be built where consumer-centered, real-time data enables the unique ability to quantify and observe population health parameters, providing potential return on investment in some disease prevention use cases. For example, most high-end consumer smartwatches include sensors for oxygen saturation, heart rate, electrocardiogram, and activity level. These data points can be useful in that they correspond to physiological parameters of severity and exacerbation risk and large and impactful public health burden [ 19 ]. The context of use of these technologies with respect to individual attitudes, socioeconomic disparities, and privacy concerns remains a work in progress.

Abad et al. [ 20 ] completed a comprehensive systematic review of digital public health surveillance (DPHS) that included 755 articles published over the past 15 years. The studies were conducted in 54 countries and used 26 digital platforms addressing 16 public health surveillance domains topped by infectious diseases and with lesser frequently, behavioral risk factors. The study uncovered a dearth of methodological and practical limitations in longitudinal studies that interfered with the integration of DPHS into public health activities as well as the synthesis of a cohesive body of RWE. Data limitations on the demographic characteristics of internet users were explicitly noted, and many studies did not stratify by geographic region. The most significant limitations identified in these studies were:

- 40% had limited sample sizes and scope attributed to limitations in manual coding and qualitative analysis;
- 80% may have had content bias;
- 85% were limited to English-language content;
- None assessed health issues in vulnerable populations;
- 41% used biased samples or samples that were not representative of populations;
- 36% did not segment results by geographic location, thus limiting the conclusiveness, contextualization, and interpretability of results;
- Only six studies showed how DPHS has been implemented in public health programs.

The authors identified significant challenges regarding the generalizability and validity of social media and internet data and cautioned against drawing inferences from these types of data for digital public health surveillance. The review provided a strong use case for improved AI, data processing technologies, and the integration of data and knowledge from multiple domains throughout the patient journey and reminded us that deficiencies in data quality, provenance, and reliability can impact us all, public health professionals, bioinformaticians, data scientists, and, clearly, patients and their caregivers.

Two reviews provided valuable insights into the status of digital health technologies in public health. In the first, Canfell et al. [ 15 ] described that aggregation and meaningful presentation of the digital health data can enable: (1) advancement from static and retrospective to accurate and real-time measurements of population health; (2) precision prevention interventions, targeting precise, at-risk groups or communities by tailoring interventions to unique characteristics; and (3) monitoring population health intervention and metrics of improvement to create deliverables that justify the necessary funding shifts to enable efficient implementation of predict–prevent models.

In the case of COVID-19, a review by Gunasekeran et al. [ 21 ] performed a systemic review of digital health applications for population-level public health responses during the first six months of the pandemic. The team used a scoping review approach to map out the range and nature of evidence to answer the fundamental question: “What forms of digital health have been applied for public health responses to COVID-19?”. The authors concluded that significant challenges remain in the adoption, scaling, and integration of precision prevention and precision epidemiology tools into healthcare systems and public health programs. Other considerations included issues related to equity in deploying precision technologies among disproportionately affected groups where technology access may be limiting. Further considerations focused on the inherent potential for measurement bias, especially for racial and ethnic minority groups – biases caused by the use of unvalidated AI models trained on nonrepresentative data sets [ 5 , 22 , 23 ]. Both reviews highlighted the challenges and urgent need for more investigations and evaluation of the validity and utility of digital platforms and AI models in precision prevention and epidemiology. There are only a limited number of studies investigating the implementation of these tools and, importantly, patients' and healthcare providers' acceptance of digital tools. Together, these gaps illustrate the need for more participatory research so that a sustained adoption of these tools beyond the pandemic can be realized.

Another example of potentially high-impact utilization of digital tools is the evaluation of pulmonary function. Spirometry is a medical test that measures how much air the patient can breathe in and out of the lungs in the form of forced expiratory volume and how fast the exhalation takes. It is the test of choice to evaluate airway dysfunction and obstruction and helps to monitor disease severity and predict future exacerbation risk. In spirometry, digital informatics can monitor symptoms and changes in lung function, which is an integral part of asthma and chronic obstructive pulmonary disease (COPD) management. The Mayo Clinic Children's Center has developed a new Mayo Clinic Pediatric Difficult to Control Asthma Clinic, which uses technology to provide comprehensive, multidisciplinary evaluation and treatment to patients with severe and difficult-to-control asthma. The clinic uses asynchronous virtual direct observed therapy (DOT) to monitor adherence to the treatment plan.

### 3.2. Societal Trends in Health Preservation and Disease Prevention

The National Center for Chronic Disease Prevention and Health Promotion (NCCDPHP) of the CDC promotes chronic disease prevention in four key domains: epidemiology and surveillance, environmental approaches, health care system interventions, and community programs linked to clinical services. These domains were designed to help achieve NCCDPHP's vision of healthy people in healthy communities and lead to a societal trend toward healthier populations. With an increase in the number of “digital citizens” [ 24 ] and increased prevalence of the “quantified self” [ 25 ] among the population, digital informatics tools are increasingly used to collect, store, and analyze health data. A digital citizen is generally defined as a person using information technology (IT) to engage in society, politics, and government. “Quantified self” refers to the practice of tracking and analyzing personal data, such as physical activity, sleep, and diet, using technology to gain insights into one's health and well-being and such that health data is often freely shared by citizens. A recent paper highlighted the utility of characterizing the exposome as part of the quantitated self (i.e., lifelong microbial, chemical, and environmental exposures) [ 26 ]. The exposome refers to the totality of all environmental exposures across one's lifetime and can be categorized into three domains: the specific external domain, the general external domain, and the internal domain. The internal domain comprises processes intrinsic to the body, including metabolic profiles, hormones, inflammation status, as well as exogenous agents that reside on the skin or are metabolized by the body. The specific external domain (the immediate local environment) includes air pollutants, noise, soil, water, and food pollutants (e.g., pesticides, disinfection by-products), lifestyle (e.g., smoking, drinking), and occupation. The general external domain contains the societal, economic, and psychological factors, such as the climate and weather, urban and natural environments (e.g., land coverage, vegetation), socioeconomic conditions (e.g., education), psychological and spiritual stress, and health plans and policies. This opens new means to study health and disease in subsets of the population. However, caution is warranted in interpreting such data as there is inherent bias when studying a self-selecting population. Also, informatics tools currently available may not allow full integration of the different types of data collected in exposome analyses.

Digital informatics can identify patterns and trends in health outcomes and inform public health interventions [ 27 ]. For example, evaluating social network data in combination with increasingly available digital healthcare data could lead to a novel, more nuanced understanding of social and behavioral variables that account for the interplay of the individual and the network about health outcomes [ 28 ]. The CDC recognizes the role of SDOH in shaping health outcomes, but the acquisition of SDOH data and its integration into clinical workflows continue to pose significant challenges [ 6 ]. Healthy People 2030 is a national initiative that aims to improve the health of all Americans by setting evidence-based goals and objectives for health promotion and disease prevention. The initiative focuses on addressing the significant health challenges facing the United States (US) today, such as obesity, heart disease, and cancer. Healthy People seeks to eliminate health disparities and improve the quality of life for all Americans, with one of its key priorities being the recognition of SDOH in shaping health outcomes.

Many national health and medical organizations recommend capturing and using SDOH information in clinical settings to improve patient care and advance population health goals. Digital health tools and platforms are evolving across multiple levels to address this goal. Nguyen et al. [ 29 ] conducted a study to understand barriers and facilitators in underserved communities associated with access to health-enhancing resources such as healthy foods and transportation technologies to build socio-technical interventions that meet residents' needs. The authors conducted in-depth interviews, participant-led neighborhood tours, and clinic visit observations involving ten patients with diabetes in underserved San Francisco neighborhoods and ten community leaders serving those neighborhoods. They then coded health barriers and facilitators using a socio-ecological framework and linked these qualitative data with early personal development, focusing on patients' experiences in these communities and within the healthcare system as a starting place for a future informatics design. Social risk and protective factors across almost every socioecological domain and level were identified — from physical disability to household context to neighborhood environment. Using multiple methodologies, the study provided a socioecological approach to understanding health promotion for patients with chronic disease in a safety-net healthcare system. Indeed, it has been argued that ambient data [ 12 ] and ambient intelligence [ 30 ] can inform design and understanding of the role of the physical environment in health-determining behaviors. Future digital health research should center on the lived experiences of marginalized patients and their providers to design and implement informatics solutions effectively for them.

An increasingly popular approach involves geocoding, an approach that allows conversion of addresses (street names or postal codes) into geographic coordinates (latitude and longitude) that can be linked to health data. This linkage allows hospitals, government registries, and other health institutions to evaluate health outcomes relative to geographic location [ 31 ], and enables targeted interventions to address disparities and improve access to care. Interestingly, geocoding and health data privacy intersect, and this juxtaposition has precipitated the buildout of governance infrastructure for data visualization in public health research and practice. The functions of this ecosystem have been harnessed for representation and data governance with genomic data.

### 3.3. State of the Art of Digital Informatics

AI and ML have advanced significantly and are increasingly being used in disease prevention and treatment. The CDC uses AI to identify patterns and model trends in health outcomes and inform public health interventions. AI and ML can be used to model and predict biological activities, discover new drugs, and guide precise disease prevention strategies based on a granular assessment of disease biology.

Precision medicine is a term often associated with the use of genetic and molecular profiling and other risk factors to optimize efficiency or therapeutic benefit for groups of patients. However, precision medicine is more than that — it is an emerging field of medical practice that relies on state-of-the-art technologies, including high throughput bioanalytic technologies like next-generation sequencing, array-based technologies mass spectroscopy, bioinformatics, and clinical decision support, all of which can be augmented by AI and ML strategies, to integrate biological, environmental, and lifestyle influences on health. Clinical informatics and biomedical informatics are two developing but essential areas for advancing precision medicine, focusing on how information is collected, stored, analyzed, and disseminated [ 32 ].

Digital informatics is rapidly evolving with the development of new technologies. This field focuses on collecting, storing, and analyzing data, and the use of these products to provide relevant insights. The term encompasses many applications, from public health to manufacturing to transportation. Digital twins are characterized by the seamless integration between the cyber and physical spaces and are used to model, simulate, and optimize complex systems in silico before incurring the costs of running experiments in the physical domain [ 33 ]. As such, a digital twin is a digital model of a physical object or system that serves as an indistinguishable digital counterpart for practical purposes, such as simulation, integration, testing, monitoring, and maintenance. Digital twin technology uses data streams to create a digital representation of a real-world asset to improve collaboration, information access, and decision-making. In healthcare, digital twins are utilized to build digital representations of healthcare data, such as hospital environments, laboratory results, human physiology, etc., through computer models [ 34 ]. Indeed, digital twin technology modeling of real-world data (RWD) has been used to build synthetic control cohorts [ 35 ]. The technology is qualified for use by the European Medicines Agency (EMA) and has been used in an interventional drug registration trial for Alzheimer's disease [ 36 , 37 ]. Researchers have recently proposed avatars as digitally rendered simulations based on patient-specific multimodal data into AI/ML models as a new paradigm coupling translational research and patient care [ 38 ].

Digital twins of the human body can also be applied for modeling organs, microphysiological systems, and single cells or an individual's genetic makeup, physiological characteristics, and lifestyle habits to create personalized medicine approaches and treatment plans. These replicas of specific aspects of the body's internal systems can improve medical care and patient treatment by providing data that serves as the basis of clinical trials and research data. Digital twin technology has the potential to revolutionize healthcare systems by leveraging real-time multimodal data integration, advanced analytics, and virtual simulations.

Disease prevention throughout the course of life remains the hallmark of health promotion and population health. Unfortunately, underrepresented communities in the US often present with more significant risks, and at later stages of disease, that predispose these populations to a greater likelihood of disease and worsened health outcomes. These challenges are perpetuated by a myriad of factors, including longstanding mistrust of the healthcare system, geographical distance from care, language barriers, and fear of encountering implicit bias and stereotyping during care, among others. Underrepresented communities are often at greater risk of not receiving optimal care and thus experiencing sub-optimal health outcomes. Traditional medical approaches focusing on the “average patient” often produce mixed results for these populations as they fail to account for underlying social, cultural, environmental, and lifestyle factors contributing to health [ 39 ]. Importantly, low participation in clinico-genomics research and databases means that genetic variants specific to these populations are generally not well characterized in clinical genetic knowledge bases [ 23 , 39 ], leading to delays in the development of clinical guidelines and the amplification of disparities.

In practice, clinically oriented laboratories and analytics companies have begun to reduce the cost of sequencing whole exomes and whole transcriptomes to a price point where complete assessment of variants is within the reach of the payer, the patient, and the clinician. Whole genome sequencing is fast becoming practical at point-of-care based on the rate of change in cost per mega-base, which is a cost reduction curve much steeper than Moore's Law, which postulates that technology reduces microprocessor costs by half every 18 months. As genetic sequencing technology continues to advance, clinical value will increasingly be created at the point of care for the delivery and interpretation (decision tools and rules) of complex genetic information using AI-assisted clinical decision-making. This is in fact the current bottleneck preventing widespread adoption of sequencing in healthcare; a friction rooted in cost but also implementation complexity [ 22 ].

AI, as applied in clinical decision-making, is already used with waveform physiological data in the intensive care unit setting [ 40 ]. Using RWD and RWE to power knowledge about populations and disease processes, we recently initiated an idealized clinico-genomics registry, unlocked by computational technology and AI, especially the blockchain technology [ 41 ]. The model is being applied to evaluate predisposition to diseases such as cancers, psychiatric afflictions, and metabolic disorders, that strongly manifest at the intersection of genetics and SDOH. Use of digital tools and virtual clinical research practices to engage cancer patients longitudinally and virtually where they live holds promise in capturing the nuances of SDOH and establish a wider perspective to address health disparities across the natural history of disease. Blockchain is a versatile technology with many attributes that are useful in addressing the challenges confronting an idealized clinico-genomics registry across time and geography [ 23 ]. The smart contracting capabilities of blockchain can provide a patient — or any official in a specimen/data supply chain with a handheld computer — the capability of allowing or denying access to a registry resource, whether an entire EHR or a single data object, which can be limited to a single genotype for a single allele for a single patient. This allows any stakeholder to expand or revoke consent with the ease of a mobile app, much like a click-through license for a piece of software or a downloaded app. The establishment of control, transparency, and agency of one's health data reduces the leap of faith required to share data and may lower trust barriers to data sharing and research participation.

**Figure 2.**
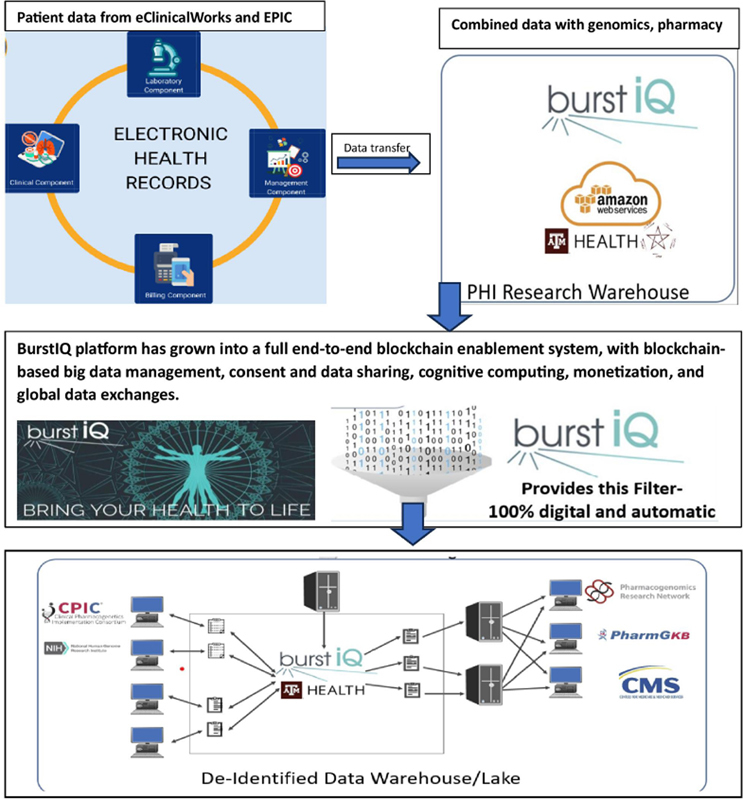
Data Follow and Strategy in TAMU Idealized Clinicogenomics Registry

Building a digital cohort using case-level data over time or across organizational boundaries entails a myriad of ethical, legal, and administrative quandaries, leaving the promise currently out of reach. An idealized clinico-genomics registry ( Figure 2 ) provides a systems perspective (with scientific rigor) on health's social, economic, genetic, and environmental determinants of health and disease outcomes. However, this level of sharing requires a leap of faith by the patient that is often too high a threshold for socially and economically marginalized populations, who perceive aspects of clinical research participation as subverting their power and autonomy or view the benefits of participation as being too abstract or diffuse. Indeed, affording patients and health systems transparency and control over their role in clinical research can be game-changing. If marginalized populations are not better represented in clinico-genomics registries, the AI tools trained on these assets will continue to amplify health disparities. There is an acute social and moral impetus to reverse the long-standing disengagement of socially and economically marginalized populations from clinical research as AI models increasingly power precision medicine delivery. Patient-centric data governance promises to give marginalized populations a proactive voice in their participation in clinical research and enable them to experience the benefits and consequences of participation directly.

Cloud computing, mobile computing, digital ledgers, tokenization, and AI technologies are powerful tools that promise to give agency to all data stewards and enhance longitudinal patient engagement across the natural history of disease. These tools also promise to enhance engagement by giving participants agency over their data and addressing a major impediment to research participation. This will only occur if these tools are available to all patients. Distributed ledger technologies (specifically blockchain) converge these tools and offer a significant element of trust that can be used to engage underrepresented minority populations more substantively in clinical research. Combined with the efforts of our multidisciplinary health facilitators, the programs using these new technologies provide agency to research subjects over their data and bring down barriers that help promote trust. This is a crucial step in linking composite cohorts at population scale for training and optimizing AI tools for enhancing public health in the future.

## 4. Discussion

To illustrate the implementation of many of these tools into concrete real-world impact, examples of different clinical applications and use of a menu of technologies were presented in this review. Advances in computational cost and throughput have enabled the combination of high dimension, multi-omics data with medical imaging to design algorithms that efficiently identify digital classifiers, discover more valuable biomarkers, and, through a systematic combination of these new technologies and traditional bioanalytic tools, ultimately narrow the latency between researchers and implementation by clinicians in precisely classifying and predicting disease processes. However, constructing novel biomarker detection algorithms and identifying and validating high-performance and robust biomarkers in the clinical setting continue to be challenging problems due to regulatory, commercial, and implementation barriers.

In addition to Healthy People 2030, several other societal trends are shaping a culture of health preservation and disease prevention. The use of telemedicine, which allows patients to receive some forms of medical care remotely, without having to visit a doctor's office or hospital has grown in prevalence due to the expansion of broadband networks, cloud computing infrastructure, and especially specific adaptations to healthcare delivery during the COVID-19 pandemic [ 42 ]. The World Health Organization (WHO) defines telemedicine as “an interaction between a healthcare provider and a patient when a distance separates the two”. COVID-19 forced dramatic shifts to telephone and video consulting for follow-up and routine ambulatory care. Short message service (“text”) messaging also proved a useful adjunct to remote consulting, allowing the transfer of photographs and documents. These options proved to be especially beneficial for individuals in rural or remote areas or those with difficulty accessing medical care due to mobility or transportation issues. A major gain in the provision of telemedicine services has been realized in mental health, as conditions such as depression and anxiety significantly impact an individual's physical health, and affect their ability to work, socialize, and engage in other activities. As a result, there is a growing focus on integrating mental health care in primary care settings, with the provision of remote mental health services that are accessible, affordable, and culturally appropriate [ 43 ]. Finally, telemedicine has provided promising applications for remote monitoring of sleep apnea [ 44 ], COPD [ 45 ], asthma self-management [ 46 ], and pulmonary rehabilitation [ 47 ].

Integration of many of the data collection modalities cited in this review have great promise in building robust and generalizable models predicting chronic disease and its progression. The cases presented below showcase the broad applicability of digital informatics in precision prevention, epidemiology, and clinico-genomics research. For instance, COPD, a chronic lung disease with a prevalence of over 16M in the US [ 48 ], represents a major public health concern and a challenge for patients, providers and payers. The CDC developed a public health strategic framework for COPD prevention that aims to improve the collection, analysis, dissemination, and reporting of COPD-related public health data, increase collaboration among stakeholders with COPD-related interests, and heighten awareness of COPD among a broad spectrum of stakeholders and decision-makers [ 49 ]. These tools are necessary, as COPD is an extraordinarily complex and heterogenous disease [ 50 ]. The pathogenesis of COPD is complex, so computational studies require robust data collection methodology and large sample sizes [ 51 ]. Chronic management and documentation of COPD utilizing wearable physiological sensors, digital spirometry, and population scale clinico-genomics represents fertile ground for implementating science around these new technologies in both clinical care and clinical investigation.

Public health informatics is also being increasingly used to monitor and improve sleep disorder diagnosis and treatment. Sleep disorders are inherently observable and tractable with data, although progress has been impeded by the traditional overnight sleep clinics used for diagnosis. Sleep-Omics is a new paradigm of sleep medicine that uses big data, sensor technology, and analytics to monitor sleep in more “natural” settings to improve diagnostics, prediction, and quality of interventions. Sleep-Omics can help identify the basic functions of sleep and how to modulate sleep as a novel clinical intervention for improving cognition, mood, and longevity. In addition, digital informatics is being used to improve the classification and diagnosis of sleep disorders, predict disease and treatment prognosis, characterize disease subtypes, improve sleep scoring via automation, and leverage nightly Positive Airway Pressure (PAP) downloads for improved adherence support and intervention [ 52 ]. As treatment options expand for sleep disorders, more multimodal data enabling precise classification of the nature and severity of the disease will likely have greater utility. Sub-phenotyping heterogeneous groups of patients according to underlying physiological endotype characteristics has been limited by the technical requirements posed by collecting in-depth respiratory and arousal data, which are then analyzed using complex signal processing algorithms. Finnsson et al. presented and validated a method for computing key physiological endotypes — loop gain, arousal threshold, and several other ventilatory parameters, showing that endo-phenotyping using the polysomnography method can be implemented in a cloud-based environment [ 53 ]. Telehealth and telemonitoring can be used to improve the classification and diagnosis of sleep disorders, predict disease and treatment prognosis, and importantly, lower barriers to obtaining and maintaining treatment.

As briefly introduced earlier, telehealth serves as a remote monitoring and delivery system of care where patient data and clinical information are routinely or continuously collected and processed, presented to the patient, and transferred to a clinical care institution for feedback, triage, and intervention by a clinical specialist. Digital informatics makes telehealth a reality. Telemonitoring involves the use of telecommunication technology to transmit data regarding patients' vital signs, symptoms, medications, and even patient-reported parameters to an operator at a monitoring station who will, in turn, transmit them to a specialist who can recommend treatment variations or new interventions to avoid deterioration in a patient's medical condition.

Telehealth and telemonitoring are increasingly being used to monitor and improve the diagnosis and treatment of pulmonary diseases. Gaveikaite et al. [ 54 ] assessed the effectiveness of telehealth solutions for improving health and health service outcomes in patients with COPD stratified according to disease severity. Some systematic reviews have evaluated the efficacy of telehealth interventions on clinical outcomes in patients diagnosed with COPD. However, the findings varied widely, and the efficacy was diverse. It may be due to lack of reporting on important patient characteristics, lack of validated data collection instruments and lack of high-quality reporting. Telehealth efficacy is impacted by the behavior of those delivering who might be resistant to new information and communication technology applications, as well as those receiving the intervention who might fail to comply. There is no doubt that the continuous evolution of digital informatics will contribute to the efficacy of telehealth.

Biomarker analyses and omics panels are increasingly being used in healthcare to improve disease prevention, diagnosis, and treatment. Public health informatics analyzes large volumes of data from these sources in rare diseases, cancer, cardiology, and pharmacogenomics, to name a few. Many data analysis methods and algorithms identifying biomarkers from omics data have been developed and applied over the last decade. These methods can work at three distinct levels: individual molecules, combinatorial features, or networks. Individual-molecule biomarker analysis is simpler and easier to perform than the other two levels. Many methods focus on identifying differential molecules in different groups as individual molecular biomarkers. Although single-molecule biomarkers are widely applied in clinical settings, they usually have a high false-positive or false-negative rate. Because of differences in platforms, samples, protocols, statistical methods, and other ancillary factors, molecular biomarkers defined by different studies may differ widely and share few common characteristics, denoting poor repeatability and reliability [ 55 ].

With the rise of increasingly powerful informatics tools ( Table 1 ), applications in public health, and new diagnostic approaches that deepen our understanding of the molecular, socio-economic, and environmental basis of disease, precision in prevention and healthcare has come within reach. Nowadays, the awareness of the potential of personalized healthcare is emerging and its impact goes beyond precision medicine to population health and epidemiology. Personalized healthcare comprises tools that allow for tailoring medical care by combining conventional clinical datasets, generalized models, biomolecular signatures (such as genetics), environment, lifestyle, and personal needs to make clinical decisions based on the specifics of a patient rather than assumptions about the average patient. Some key innovations in this area include digital healthcare solutions with technologies connecting digital patient information/EHRs with wearable devices, mobile apps, telehealth, and digital assistants using AI. Digital twins and avatars remain aspirational, but more integrated data ecosystems, interoperability, and more facile data governance provide great promise.

**Table 1.** List of major digital informatics tools that have significant applications in public health and precision healthcare.

Digital Informatics Tool (precision-based)	Explanation
Ambient Data	Information/data collected by ambient intelligence systems within healthcare settings. These systems use contact-based wearable devices and contactless sensors to gather data such as audio and imaging of physical spaces
Artificial Intelligence (AI)	An umbrella term that covers various technologies, including machine learning, deep learning, and natural language processing (NLP)
Biomarker Analyses	Biomarkers are measurable indicators of biological states or conditions. Biomarkers can be molecular, histologic, radiographic, or physiologic characteristics used to examine normal or pathogenic processes, or responses to therapeutic interventions
Block Chain Technology	A digital ledger system that uses a distributed database shared across a network of computers to record transactions. Each transaction is recorded in a block and linked to the previous block, creating a chain of blocks — hence the name “blockchain”
Electronic Health (eHealth)	eHealth refers to healthcare services supported by digital processes and technologies. It encompasses a range of services such as electronic prescribing, telehealth, and EHRs
Digital Public Health Surveillance (DPHS)	Use digital data, especially from social media and other internet-based sources to monitor and analyze public health-related trends and occurrences
Digital Twin	A virtual model that mirrors a real-world object, system, or process. It is designed to be an exact digital counterpart for various purposes such as simulation, testing, monitoring, and maintenance
Machine Learning (ML)	ML enables systems to learn and improve from experience without being explicitly programmed. It uses data and algorithms to simulate the way humans learn, gradually increasing its accuracy
Mobile Health	Use mobile and wireless technologies to support the delivery of health services and management of patient care
Omics Panels	Omics Panels in healthcare refer to comprehensive tests that analyze various biological molecules to provide insights into a person's health at a molecular level. These panels can include genomics, proteomics, metabolomics, and more, and are used for disease diagnosis, prognosis, and guiding personalized medicine
Predictive Analytics	Use data, statistical algorithms, and machine learning techniques to forecast future health outcomes. It helps healthcare organizations customize treatments, anticipate patient needs, and run more efficiently by analyzing current and historical data to predict future trends and patient outcomes
TeleHealth and Telemonitoring	Delivery of health-related services and information via telecommunication technologies. It includes a wide range of services, such as virtual consultations and health education

Several implementation challenges will continue to impede the widespread use of RWD and RWE. Certain data standards are nearly impossible to implement across diverse geographies and austere clinical environments. Current tools for managing data governance and data provenance tools do not enable full transparency among data stewards, patients, and researchers. The costs and risks associated with storage and the sharing of such data undermine clear business cases and return on investment, so harmonization of private and public investments for population scale data commons remain out of reach in most domains, though recent business model innovations in genomics and RWD from medical records have accelerated progress. These challenges impede the creation of population scale data commons necessary to achieve the promise of precision epidemiology and the deployment of the tools of genomic medicine in public health. Addressing health disparities that have roots in AI and other data driven domains is a moral imperative at this time. Otherwise, disparities will continue to widen as marginalized populations refrain from sharing data and from participating as research partners in medical and public health innovation. We posit that giving patients access to technology, data transparency, and data agency are as important as access to providers to narrow the disparities that would otherwise be magnified with the data driven medicine of the future.

The successful implementation of public health informatics, precision epidemiology and precision medicine initiatives require professionals who are not only well-versed in informatics but also professionals who share an understanding and appreciation of the realities of implementation. A report in the Journal of Applied & Translational Genomics [ 56 ] took a closer look at strategic plans focused on research and clinical implementation of precision medicine efforts. The study revealed significant gaps in professional expertise that impact the ability of health systems to relate genomic data and SDOH — reminding us that having the data is necessary but certainly not sufficient. An “informatics workforce that has interdisciplinary training to combinatorial science, applied research and clinical implementation” is needed. Indeed, medical informatics has become a quasi-specialty in medical practice as evidenced by the fact that many chief medical informatics officers are physicians. Another prevalent educational skills gap affecting precision healthcare comes from a lack of expertise in genomic research among physicians. While genomic testing and research have proliferated in recent years, physicians' expertise in this area has not increased [ 57 ] — another example of data being necessary but not sufficient to implement the best new tools available for medical practice. People, data, tool systems, policy, and legal/ethical regulations are the five vital components required for optimal integration of precision medicine. This integration is a systems design and engineering problem as much as it is a biological problem. Focusing on the people aspect, this chapter advances major aspirations for the future of digital informatics. It calls for increased employment of specialists in the technology of precision medicine and for changes in the training of medical students and specialists in digital informatics.

## 5. Conclusions

The primary purpose of this narrative review was to provide a broad landscape assessment of the field and to distill these considerations within the context of the author's perspectives. Many new concepts and new digital informatics technologies have been applied in public health and epidemiology for precision prevention. However, digital informatics in public health and precision medicine needs more input and interactions from physicians and engineers to be able to deliver meaningful advancements. The research gaps identified reflect multiple aspects in a fast-paced changing healthcare ecosystem. One of the priority areas for future investment is the training of the next generation of caregivers and the creation of innovative tool systems, policy, and legal/ethical regulations.

Furthermore, the creation of a data consortium that can facilitate expanded data access for researchers needs to be considered. Validation and evaluation of the different tools will identify shortcomings in need of further improvements, a cycle that involves an iterative process of developing, testing, and refining the technology. There is no exception in precision health. As AI and other digital informatics tools continue to advance, their integration into healthcare and public health will continue to profoundly transform the current landscape. A major requirement and challenge for the next era of health data is the protection of relevant sensitive patient data and patient rights [ 58 ]. The idealized clinicogenomics registry should demonstrate the capacity to mitigate risks and improve patients' power and autonomy making explicit the tangible benefits of participation in contributing to a population-scale data ecosystem.

Public health informatics and precision epidemiology are exciting and growing practices that can improve public health outcomes. New and AI-powered digital tools and technologies offer increasing potential for substantial benefits to medicine and public health, and their applications to public health surveillance and outcomes are likely to continue to grow. Data collection, data quality, population scale curation, privacy and data governance are challenges in multiple domains of commerce and life. The solutions being developed to advance data science in these domains are likely to be generalizable to challenges in the public health domain to the extent the public health community of practices deepens connectivity to the technology and its stakeholders.
